# Role of Gambling in Payback Failure in Consumer Credit—Data from a Large Body of Material Regarding Consumer Loan Recipients in Sweden

**DOI:** 10.3390/ijerph17082907

**Published:** 2020-04-23

**Authors:** A. Håkansson

**Affiliations:** Department of Clinical Sciences Lund, Psychiatry, Faculty of Medicine, Lund University, 221 00 Lund, Sweden; anders_c.hakansson@med.lu.se

**Keywords:** problem gambling, gambling disorder, consumer credit, loan, indebtedness, behavioral addiction

## Abstract

Indebtedness is associated with poor health outcomes, and problem gambling may contribute to indebtedness through consumer credits related to gambling expenses. The assessment of consumers’ applications for loans may be an opportunity to detect and prevent further problem gambling. The present study analyzed a number of variables including gambling-related transactions and their association with payback failure in 48,197 loans to 20,750 individuals in Sweden. Sums and frequency of gambling deposits or withdrawals generally did not predict failure to pay back loans. Instead, having a loan defaulted at some time was associated with a baseline pattern describing a theoretical loss-of-control gambling pattern (short-term intense gambling), with a higher ratio of gambling deposits or withdrawals per occasion, and with several instances of gambling in close association with a loan. While several group differences were modest, signs of rapid, short-term and intense gambling, rather than gambling itself, may identify risk of payback failure and risk of indebtedness. Implications for early problem-gambling detection and prevention, such as by gambling operators and financial institutes, are discussed and may promote better public health in relation to gambling indebtedness.

## 1. Introduction

During the past decade, an increasing number of social policy and medical research papers have addressed the potential hazards of over-indebtedness in the population [1,2]. Over-indebtedness, characterized by the inability to fulfill one’s financial obligations, has been shown to be associated with both physical and mental health conditions [3], even including suicidal ideation [4] and completed suicide [1]. Other health hazards associated with over-indebtedness include obesity [5], pain [6,7], sleeping problems and sleep medication use [8], psychosis, diabetes [9], and poor adherence to prescribed medication [10].

Being in debt, even when related to more traditional loans such as house mortgages, is associated with poor health [11]. In addition, beyond the challenges associated with traditional borrowing, modern technology also facilitates rapid transactions and may accelerate the development of debt. Payday loans have been shown to be typically short-term in their nature, such that they rapidly increase short-term liquidity but contribute to increased indebtedness over time [12], and credit card debts have been shown to be associated with poor health [13]. It is clear that payday loans are unevenly distributed across groups with differing financial status in the population; such short-term credits are more commonly used by individuals in financial difficulties, and even more so during societal financial crisis [14]. Smaller but typically rapid loans may be used in order to make up for financial difficulties, to pay short-term expenses or may contribute to a vicious circle when used for the payment of other debts and interests [15], and consumer credit may be associated with and may enhance social disadvantage [16]. It has also been shown that short-term loans correlate with a range of health problems, including both symptoms of poor physical and mental health [17]. In the setting studied here, the enforcement authority has called for increased attention to the increasing number of unsecured loans [18]. An increasingly high household indebtedness has also been suggested to have major negative impact on the country’s economy, as stated by the Swedish central bank [19]. 

Theoretically, improved debt prediction could be favorable in order to prevent individuals from indebtedness. Relatively recent attempts have been made to predict indebtedness using a large data collection [18]. Despite this, the area of prediction of indebtedness can be considered a very recent topic of research, rarely addressed in the context of public health and behavioral medicine.

Gambling is a phenomenon inherently associated with a risk of losing money and developing debts, and gambling disorder is a psychiatric diagnosis listed among the addictive disorders in the diagnostic manuals of the American Psychiatric Association [20] and the WHO diagnostic classification [21]. Gambling disorder is thereby the first, and the most clearly established, addictive disorder described as behavioral, i.e., not including the intake of a substance [20,21]. It is reasonable to assume that problematic gambling contributes to a non-negligible proportion of indebtedness in society. Financial difficulties are among the well-known consequences of problem gambling and contribute to the fulfillment of diagnostic criteria of a gambling disorder [20], and it has been shown that debts contribute to a significant part of the psychological distress associated with problem gambling [22]. Problem gambling across most studies and settings has been described to affect 1–5% of the general population [23], and the diagnostic construct of gambling disorder describes a severe condition, associated with high psychiatric comorbidity [24] and an increased risk of suicide death [25]. In the geographical setting addressed in the present study, the clinical picture of gambling disorder is known to have changed, with a marked predominance of online gambling among patients presenting with gambling-related conditions [26]. Modern online gambling promotes highly short-term gambling, such as in-play betting on sports [27] and online casino gambling [28], and concerns have been raised about consumer credit associated with gambling [29]. One key feature of the development of a gambling problem is the gambling pattern known as loss of control, i.e., a pattern where gambling in the short term surpasses the level or frequency initially intended by the gambler, and which may potentially constitute an important share of the problem picture in gambling [30].

Problem gambling is a public health challenge, with a great need for improved early detection. The early detection of problem gambling is difficult, and based on the close connection between problem gambling and debts. Loans may present a window of opportunity for the possibility of identifying problem gamblers. Thus, theoretically, the use of consumer data regarding financial transactions, including those associated with gambling, may present an opportunity for public health-oriented intervention. Therefore, the present study aims to analyze, in a large dataset of loans, whether measures of specific gambling patterns can predict subsequent failure to fulfill re-payment of loans. Based on the addictive features of gambling, it was hypothesized that, in a large sample of individuals taking loans, payback failure would be predicted by a prior pattern of: (1) more extensive, more frequent or more intense gambling involvement; (2) the close association of loans and gambling, described as any gambling occurring prior to or after a loan; or (3) a higher degree of gambling featuring a ‘loss of control’ pattern of rapid and intense short-term sequences of gambling.

## 2. Methods

The present study is a statistical analysis of a large dataset of loans and bank account transactions, including gambling-related transactions during the period prior to the loan taken. In the data collection, the author has collaborated with the company Instantor AB (www.instantor.com), a Sweden-based company offering models for risk management to banks and similar financial institutes who offer loans. The present study was reviewed by the national ethics board of Sweden (file number 2019-04410). The study was not judged to be applicable to national ethics legislation, as it does not involve any data which can be directly or indirectly identified. The study involves fully anonymized data (i.e., without any personal data) held by Instantor AB, which, after the ethics procedure, provided the researcher with the dataset for statistical analyses and scientific interpretation. 

### 2.1. Setting

The present study is carried out in Sweden, a setting where the age limit for consumer credit and other loans, as well as for gambling, is 18 years. The gambling market in Sweden was—during the period of time from which the present study originated—formally regulated by an oligopoly system. In this system, a state-owned monopoly was responsible for a large number of gambling activities, whereas a limited number of operators were allowed to administer lotteries, of which several lotteries were associated with charity organizations, and horse racing. In this gambling market, online casino games were formally non-existent (and land-based casinos were among the most regulated gambling activities, with four state-owned establishments in four major cities in the country). However, in recent years, overseas online gambling operators, offering mainly online casino and online sports betting, strongly increased their market share, and among treatment-seeking patients at a gambling disorder facility from 2015 to 2016, a large majority of patients reported online gambling as their principal type of problematic gambling, with online casino (despite its unauthorized status in the country) being by far the most common gambling activity [26]. Also, overseas online gambling operators, mainly in the area of online casino, constituted a majority of the large share of gambling companies in television advertising during the period [31]. New legislation, from January 1st 2019 [32], in place after the end of the data collection for the present study, changed the gambling market into a license-based system allowing a large number of gambling operators, including online casino operators, under the condition that they follow specific responsible gambling measures, novel regulations for advertising gambling, and adherence to a national self-exclusion system for gambling. 

### 2.2. Participants

The present study is based on data from residents in Sweden above 18 years of age (as loans to minors are not allowed). The dataset is based on loans applied for and granted to individuals from a financial institute (fully anonymous, i.e., not including any kind of personal data, including data given to the author, in order to protect full confidentiality of the data) during the years 2015–2018. These individuals had one or several loans during the study period. For the sake of limiting the data processed, the only specific category of transactions reported to the author was that of gambling-related transactions. The present study also involves the amount of the loan granted, self-reported income, debts and an estimation of the individual’s monthly expenses, as well as transactions made to and from the individual’s account prior to and after the loan. 

For gambling data, registered expenditures include the deposit of money from an individual’s bank account into to any account of a gambling operator; thus, one such deposit may be used for several subsequent gambling occasions within that operator’s account. Likewise, a gambling-related income registered in the study incudes a withdrawal made from a gambling operator account to the individual’s private account. Thus, a gambling win used for further gambling, and therefore kept and used within the gambling account at the gambling operator’s site, is not registered as a gambling withdrawal (or expenditure) in the present study. 

### 2.3. Measures

In one part of the study, only one loan (the first loan registered during the study period) for each person was analyzed. 

Outcome Measures:

Whether or not a specific loan was either defaulted (i.e., the full amount was never paid back) or defaulted and later recovered (defaulted/recovered). In order to simplify data reporting, the associations with loans being defaulted were not reported, and only associations of defaulted/recovered loans were reported. 

Independent Variables

Individual characteristics:

- Self-reported monthly income, self-reported monthly spending, and self-reported total debt (all in SEK).
- Granted amount (in SEK), which may have differed from the amount applied for.

Extent and frequency of gambling involvement:

- Frequency of gambling expenditures (deposits) and gambling withdrawals during the past 30, 90, and 180 days, respectively.
- The total sum of gambling expenditures (deposits) and gambling withdrawals during the past 30, 90, and 180 days, respectively.

Intensity of gambling involvement per occasion:

- The ratio of gambling deposits and withdrawals per occasion (the total sum of gambling expenses divided by the number of gambling expenses, and the total sum of gambling withdrawals divided by the number of gambling withdrawals), during the past 30, 90, and 180 days, respectively (reported for the sub-group of individuals with any gambling deposits or withdrawals, respectively, during each time period).

Gambling in association with loans:

- Whether or not the individual had a transaction history including gambling expenditure during two days prior to, or during two days after, taking a loan, during the past 30, 90, and 180 days, respectively.

Gambling patterns indicating possible loss of control:

- The existence, and frequency, of a theoretical loss of control (LOC) during the past 30, 90, and 180 days, respectively, and the number of days in loss of control during the past 30, 90, and 180 days, respectively. LOC was defined as having made at least three deposits to gambling companies over a consecutive three-day period, for a total amount of at least 1000, 3000, 5000 or 10,000 SEK, respectively, at any time during the past 30, 90 or 180 days.

In the second part of the study, each individual was analyzed with respect to whether characteristics surrounding the first loan predicted the inability to pay back any of the loans of that individual during the study period.

Outcome Measures:

Whether or not any of the individual’s loans were: (1) defaulted; or (2) either defaulted or defaulted and later recovered (defaulted/recovered).

Independent Variables

Individual characteristics:

- Self-reported monthly income, self-reported monthly spending, and self-reported total debt (all in SEK) for the first loan of the study period.
- Granted amount (in SEK, which may have differed from the amount applied for) for the first loan of the study period.

Extent and frequency of gambling involvement:

- Frequency of gambling expenditures (deposits) and gambling incomes during the past 30, 90, and 180 days prior to the first loan of the study period, respectively.
- The total sum of gambling expenditures (deposits) and gambling incomes during the past 30, 90, and 180 days prior to the first loan of the study period, respectively.

Intensity of gambling involvement per occasion:

- The ratio of gambling deposits and withdrawals per occasion (the total sum of gambling expenses divided by the number of gambling expenses, and the total sum of gambling withdrawals divided by the number of gambling withdrawals), during the past 30, 90, and 180 days prior to the first loan of the study period, respectively (reported for the sub-group of individuals with any gambling deposits or withdrawals, respectively, during each time period)

Gambling in association with loans:

- Whether or not the individual had gambled during two days prior to, or during two days after, taking a loan, during the past 30, 90, and 180 days prior to the first loan of the study period, respectively.

Gambling patterns indicating possible loss of control:

- The existence, and frequency, of a theoretical loss of control (LOC) during the past 30, 90, and 180 days prior to the first loan of the study period, and the number of days in loss of control during the past 30, 90, and 180 days, respectively. LOC was defined as having made at least three deposits to gambling companies over a consecutive three-day period, for a total amount of at least 1000, 3000, 5000 or 10,000 SEK, respectively, at any time during the 30, 90 or 180 days prior to the first loan of the study period.

At the end of the study period, one Swedish Krona (SEK) corresponded to 0.11 USD or 0.10 Euros.

### 2.4. Statistical Methods 

For all comparisons, individuals with defaulted loans were compared to individuals whose loans were paid back, and defaulted or defaulted/recovered loans were compared to individuals with loans neither defaulted not recovered later. Categorical data were analyzed using chi-square tests, and continuous data were analyzed using the Mann-Whitney U test. Due to the frequent skewness of continuous data, this was reported for each group using the median, and the 90th, 95th and 98th percentiles, respectively. Despite the risk of skewed data, a one-way ANOVA was also calculated for control for the continuous variables. As the total sum of gambling deposits/withdrawals and the monthly income and reported total debt were variables with very high skewness including outlier data, the ANOVA was not calculated for these variables. Statistically significant associations were defined as the p value being lower than 0.05. 

## 3. Results

A total of 20,750 unique individuals were included in the study, with a total of 48,197 loans (thus, 20,750 represent the primary loan occasion during the study period of unique individuals, and the remaining 27,447 are subsequent loans to the same individuals). The median of loans per individual was one (inter-quartile range 1–3, 90th, 95th and 98th percentiles 5, 7 and 10, range 1–52). During the past 180, 90 and 30 days, respectively, 42, 36 and 29% of individuals had had any gambling expenditure. Baseline characteristics of the participant are shown in Table 1.

### 3.1. Analyses of All Loans 

In the analysis of all loans (i.e., one or several loans for the same individual), the risk of a loan being defaulted was higher in primary loans (the first loan during the study period, 19 vs. 7%, p < 0.000001) than in subsequent loans and, likewise, the risk of a loan being defaulted/recovered was higher in primary loans (31 vs. 13%, p < 0.000001). Forty-four percent of the loans (n = 27,218) were associated with any gambling expenditure during the past 180 days. Among all loans, the risk of a loan being defaulted was the same in cases associated with or not associated with any 180-day period of gambling (12 vs. 12%, p = 0.79), and the risk of a loan being defaulted/recovered was also the same (21 vs. 21%, p = 0.98). The risk of a loan being defaulted (p = 0.36) or defaulted/recovered (p = 0.023) was unrelated to the number of 180-day gambling expenditures. 

#### 3.1.1. Analyses of Primary Loans (Primary Loan of Each Study Subject)

Primary loans associated with any period of 180-day gambling were equally likely to be defaulted, compared to individuals with loans not associated with gambling (19 vs. 19%, p = 0.15, n = 20,747), and nearly significantly less likely to be defaulted/recovered (30 vs. 31%, p = 0.06, n = 20,740). 

The risk of a loan being defaulted/recovered was unrelated to the loss of control measures for 1000 and 3000 SEK for 30 days, and for 1000, 3000 and 5000 SEK for 90 days and 180 days, whereas the loss of control measure was more common in defaulted/recovered loans for 5000 SEK for 30 days (9 vs. 8 percent, p = 0.009), 10,000 SEK for 30 days (6 vs. 5%, p = 0.003), 10,000 SEK for 90 days (9 vs. 7%, p = 0.002), and 10,000 SEK for 180 days (11 vs. 9 percent, p = 0.005). The risk of a loan being defaulted/recovered was not higher in case of gambling prior to or after a loan, and it was even less likely to involve 30-day gambling prior to a loan (13 vs. 14 percent, p = 0.015), past-90-day gambling prior to a loan (19 vs. 21 percent, p = 0.008), or past-90-day gambling after a loan (19 vs. 21 percent, p = 0.009).

A loan being defaulted/recovered was significantly associated with a lower number of gambling deposits, both for 30 days (p = 0.044), 90 days (p = 0.023) and 180 days (p = 0.027), and unrelated to the total sum of gambling deposits, across all three time frames. A loan being defaulted/recovered was associated with a lower sum of total gambling withdrawals, both for 30 days (p < 0.001), 90 days (p = 0.001) and 180 days (p = 0.002), as well as with a higher granted amount (p < 0.001), a lower reported income (p = 0.012), lower reported spending (p < 0.001) and a lower reported debt (p < 0.001). For individuals with any gambling deposits, the risk of a loan being defaulted/recovered was significantly associated with higher ratios of gambling deposits per occasion (for 30, 90 and 180 days, all p < 0.001), and for individuals with any gambling withdrawals, the risk was associated with higher ratios of gambling withdrawals per occasion (for 30, 90 and 180 days, all p < 0.001).

#### 3.1.2. Primary Loans as Predictors of any Loan (Primary or Subsequent) Being Defaulted

A total of 5225 individuals (25 percent) had a loan defaulted at least once during the study period. (For all potential predictors of defaulted loans, see Table 2.) The total number of loans was significantly lower in individuals with defaulted loans. The risk of having a loan defaulted at some time was unrelated to the number of gambling deposits or gambling withdrawals, for all time periods, and unrelated to the total sum of gambling deposits and, for two of the times frames, unrelated to the sum of gambling withdrawals.

In the subgroups of individuals with any gambling deposit or any gambling withdrawal, for all time intervals, individuals with a defaulted loan at some time had higher ratios of gambling deposits and gambling withdrawals per occasion at their primary loan, and were more likely to have a gambling deposit after a loan. In addition, having gambled prior to a loan during the past 180 days was significantly associated with having any loan defaulted, while no such statistical association was seen for the 30-day or 90-day time frame. The risk of having a defaulted loan was higher for all measures of loss of control (except for only a marginally significant association with the number of days in the lowest amount category for the shortest period of time). Having a defaulted loan was also significantly associated with a higher granted amount, lower income, lower reported level of debts, and lower reported level of spending (Table 2). 

#### 3.1.3. Primary Loans as Predictors of any Loan (Primary of Subsequent) Being Defaulted/Recovered

A total of 8390 individuals (40 percent) had a loan defaulted/recovered at least once during the study period. (For all potential predictors, see Table 3). The total number of loans was significantly lower in individuals with defaulted/recovered loans. The risk of having defaulted/recovered loans was unrelated to the number of gambling deposits for all periods and with the number of gambling withdrawals during the past 90 or 180 days, whereas the risk was associated with a significantly lower number of gambling withdrawals during the past 30 days. The risk was unrelated to the total sum of gambling deposits and, for two of the times frames, unrelated to the sum of gambling withdrawals.

Having a defaulted/recovered loan was significantly associated with higher ratios of gambling deposits and withdrawals per occasion, for all time periods studied. The first loan was significantly more often related to having gambled immediately prior to or after a loan at any time during the past 180 days, whereas the risk was not significantly related to gambling prior to or after a loan during the past 30 or 90 days. Having a defaulted/recovered loan was significantly associated also with loss of control, for all time frames and monetary values. Moreover, the risk was significantly associated with a primary loan associated with a significantly higher granted amount, significantly lower reported level of debts, and lower reported level of spending, whereas the risk was unrelated to the level of income (Table 3). 

## 4. Discussion

The present study used a large dataset of fully anonymized data from individuals taking a loan, and analyzed whether gambling-related measures can identify the risk of the loan not being paid back, with the aim of using gambling-related variables for the prediction of indebtedness and as a potential window of opportunity for the detection of problem gambling. One finding was that, in this population of individuals applying for loans, gambling habits were seen to be very diverse and highly skewed, ranging from none to very high levels of gambling. Also, it was demonstrated that the sole existence of any gambling deposit during the period prior to the loan was not a predictor of payback problems; importantly, a measure as simple as the frequency of actual gambling deposits turned out to be a poor predictor of payback failure and even had a somewhat paradoxically negative association with the outcome. Instead, a number of more complex gambling-related measures, hypothesized to indicate a more intense and potentially problematic gambling pattern, demonstrated an association with failure to pay back. Although the differences in these measures were modest overall between individuals with or without defaulted loans, the variables associated with defaulted loans were theoretical loss of control measures (repeated and large gambling deposits within consecutive days). In individuals with any gambling during the period prior to a loan, the size of deposits or withdrawals per occasion, i.e., the sums divided by the numbers of occasions, were positively associated also with a risk of payback failure. Altogether, these hypothetically problematic gambling measures were predictors of payback failure, whereas the pure existence of gambling or the numbers of gambling occasions or sums were not. Overall, the study calls for further research focus on societal prediction measures aiming to identify patterns of gambling which represent the more intense and addictive patterns of gambling.

### 4.1. Potential Predictors of Defaulted Loans

As hypothesized, a number of gambling-related measures were more commonly detected in individuals with defaulted loans than in the remaining individuals, although differences in absolute numbers were modest. Thus, given the study hypotheses, payback failure was: (1) not clearly associated with the sole frequency and extent of gambling, but clearly and positively associated with the intensity of gambling involvement; (2) not consistently associated with measures of gambling in close relation to loans; but (3) associated with a broad range of the theoretical loss of control measures.

One consistent finding was that the frequency of gambling deposits—or wins—was not a positive predictor of payback failure. Thus, rather than the extent or frequency of gambling by itself, the intensity of gambling involvement, and the defined loss of control measures, were associated with payback failure. Loss of control measures were significantly more common in individuals with defaulted loans. Another measure of interest analyzed in a narrower group of the dataset, i.e., only among gamblers in each of the time periods analyzed, was the level of gambling deposits or wins calculated for each occasion, i.e., the ratio between sums and numbers of occasions, describing the intensity of the gambling involvement. Thus, relatively less frequent but more pronounced gambling involvement was a risk factor, along with short-term intensity in gambling defined here as a likely loss of control. In addition, having gambled in close association with previous loans was another risk factor in some analyses.

All these results support the future use of more specific and more complex gambling measures than only the occurrence of deposits or wins, as measures for the detection of risky financial situations and the risk of indebtedness. Here, it should be noted that the lack of a positive association between payback failure and frequency of gambling applies only to this type of population, i.e., among clients applying for a loan, and does not mean that the frequency of gambling does not predict gambling problems in the general population. Rather, in the overall population, a link between more extensive gambling and gambling problems can be seen as intuitive.

Loss of control is known to be a key factor in the development and clinical presentation of an addictive disorder, including a gambling disorder [30,33]. In problematic gambling behavior, loss of control could be seen as characterized by repeated gambling deposits over a limited amount of time, assumed to represent a higher wagered amount than planned. The opposite pattern of gambling, representing a low level of loss of control, would theoretically be a pattern where money is wagered sporadically on separate and distinct occasions, and where one wager is not soon followed by a new one. One particular feature of problem gambling is known to be ‘chasing’ behavior [20], most typically characterized by an urge to continue gambling in order to ‘chase’ back losses, although the ‘chasing’ of an enhanced experience after a win can be possible [34]. Altogether, chasing behavior can be suspected to explain or contribute to gambling behavior with higher short-term intensity. Although loss of control is difficult to measure fully in a non-identified dataset, the proxies chosen to represent this behavior in the present study were the repeated gambling deposits (at least three) above certain amounts of money on three consecutive days. For all monetary levels, and for all time periods, the existence of such a loss of control measure was significantly associated with having defaulted or defaulted/recovered loans, again despite the lower number of total loans in this group and despite the lack of an association between any gambling deposit frequency and payback failure.

Having gambled in close proximity to a loan may also be seen as an indicator of a loss of control behavior, or a behavior where gambling is either seen as a potential way to solve problems, or where loans are applied for in order to compensate for gambling-related loss. Thus, again, these measures indicate symptoms which are key elements in gambling disorder diagnosis, such as the ‘chasing losses’ criterion, one of the key features of gambling disorder diagnosis [20]. Again, these symptoms, related more to specific expressions of gambling-related problems, may predict payback failure in a better way than the extent of gambling in itself. These associations, however, were low in absolute numbers and non-consistent, with somewhat stronger associations for the longer time period, and more so for gambling deposits after a loan. While a link between gambling and taking a loan may seem intuitive [22], there is little research in the temporal association between credits and gambling, and here further in-depth longitudinal research, including into identified individuals, may be needed. 

The present study has a number of potential implications for risk assessment and the potential identification of problem gamblers at risk of severe complications. Thus, again, although differences in absolute numbers were modest, the short-term repetition of gambling deposits above specific levels were a better marker of payback failure than gambling in itself and calls for the identification of risk assessment in gamblers who repeat large deposits within few days. Although a narrow part of the scientific literature so far, the detection of problem gambling in consumer credits has been suggested. Sacco et al. recently demonstrated that if screening for gambling is introduced among consumer credit counsellors, they are likely to identify a larger group of individuals with gambling problems than in the general population. Feasibility and acceptability by the counsellors were also demonstrated in this study [35], which represents a novel area of research and methodological work aiming to better detect and intervene in early problem gambling. The present findings strengthen the rationale behind such efforts and suggest that particular financial data may be involved in such an active screening approach. In this area, considerably more research is needed, not only in the consumer credit market, but also in more specific samples of gamblers or gamblers in specific types of gambling modalities.

In addition to the setting studied here, i.e., individuals receiving a loan, the present findings also call for the identification of high-risk patterns of gambling deposits in other settings, such as by gambling operators or in clinical risk assessments. Although the present study is not conducted in a treatment or mental health setting, the uptake of loans in such a setting could potentially be a situation where people at risk of gambling problems could be identified. Research is increasingly suggesting too the gambling industry itself as an active part in the identification of and intervention in problem gambling [36]. In line with the present research, a potential intervention by a gambling operator could be to identify loss of control measures with the aim of helping an individual reduce or give up gambling, most likely, though, in the context of a non-profit-driven responsible gambling practice. 

It should also be borne in mind that the population analyzed here is likely to be a population with an overall very intense pattern of expenditure, in other areas than gambling, which may contribute to the relatively limited magnitude of differences between individuals with or without payback failure. These expenses may include many types of shopping or other financial transactions. Shopping habits, including compulsive shopping, can be assumed to contribute to an unknown extent to short-term borrowing in society [37]. Likewise, it can be assumed that shopping patterns and gambling patterns may partly overlap; a recent web survey study in the present setting examined health and behavioral correlates of problem gambling and demonstrated an association between problem gambling and symptoms of addictive shopping [38]. Addictive shopping, or compulsive shopping, presents a relatively well-documented pattern of symptoms, and although hitherto not recognized as a separate diagnostic construct, this condition has been increasingly outlined in the scientific literature in recent years [39]. In this specific population of individuals applying for loans, shopping behaviors may need to be addressed, over and above the public health interest in problem gambling, and more research is needed in order to understand how these factors may be related to one another. 

### 4.2. Characteristics of the Present Population

Importantly, when assessing the present sample of individuals applying for loans, gambling expenses in part of the population appear to be very high. Thus, the population studied here could potentially be assumed to have a more intense gambling pattern than does the average general population. Few population studies are available for adequate comparisons, but two studies with relevance for the Scandinavian context can be referred to for some comparison. In a Danish general population survey, with respondents recruited to a web survey on gambling habits, 80% of respondents had a monthly gambling loss of around 150 SEK or less, compared to a past-30-day gambling expenditures of 150 SEK or less for only around 26–28% in the present dataset. With the same comparison, 0.9% of the general Danish population had a monthly gambling loss of more than 1500 SEK, compared to a 16–18% prevalence of a past-30-day gambling expenditure above that amount in the present study. In the present dataset, almost one out of ten participants had a past-30-day gambling expenditure of more than 10,000 SEK (data not shown), which corresponds to the gambling loss of roughly 0.1% in the Danish general population study [40]. Likewise, one Swedish study, conducted into online gamblers specifically, can be used for comparison and could be suspected to include a high-risk population with respect to gambling problems [28]. In the present study, around 28% had a past-30-day gambling expenditure of more than 100 SEK. Among moderate-risk and problem online gamblers in the online gambling study recently conducted in Sweden, the percentage reporting a past-month loss of more than 100 SEK was 85%. However, in the sample of moderate-risk or problem online gamblers, 6% had a monthly loss of more than 10,000 SEK [28], compared to a past-30-day gambling expenditures of more than that amount in around 8–10% in the present study. Thus, like in the Danish study, the percentage of high-intensity gamblers in the present population appears to have a more extensive gambling pattern than in the general population, although less extensive than in a specific online gambling population. In summary, the present sample can be described to be a population with a moderate prevalence of any gambling, which may not be a higher prevalence than in the general population, but with a sharply skewed distribution of gambling data, with a relatively large percentage of individuals reporting very high or even extreme gambling amounts. 

## 5. Strengths and Limitations

The present study is an explorative study in a type of dataset and in a scientific issue rarely included in quantitative behavioral research. Therefore, the statistical analyses were reported only as non-adjusted analyses comparing individuals with or without failure to pay back their loans, and these analyses can therefore be described as explanatory. In light of the scarcity of available variables in the current situation, involving a loan application, it also would not be intuitive to choose which variables to control for. A large number of comparisons were made for different gambling levels in different time frames, such that a certain risk of mass significance cannot be excluded. Regarding statistical analyses of gambling data, in addition to the Mann-Whitney U tests, ANOVA calculations were also added, when judged to be appropriate, and largely confirmed the same statistical associations, although with this method specific LOC measures in the lower range of monetary levels were not significant. In addition, despite an extensive analysis of transactions defined as gambling-related, it is difficult to exclude that a transaction could have been made to a gambling operator without being detected here. However, although difficult to quantify, such a difficulty of defining actual gambling operators has been judged to be limited.

One major limitation of the present study is that, due to issues of confidentiality, no identifying data, including gender and age, could be included in the analyses. Likewise, gambling behavior could be quantified only from objective deposit and withdrawal data, whereas individualized information about whether a gambling disorder diagnosis was fulfilled or not, could not be studied. Gender is known to be of relevance to the field of gambling, as male and female gambling patterns and rates of gambling problems are known to differ [41,42]. In recent years, however, more traditional patterns with a large male predominance may be altering, as in the present setting, and recent data have documented that the risk of problem gambling in women may be comparable to that of men among online gamblers [28]. Although a necessity due to the confidentiality concerns applied in the present project, future research in smaller samples with active inclusion and consent procedures may need to include socio-demographic data to control for, such as gender and age data, as well as structured assessments of the degree of problem gambling or the number of diagnostic criteria fulfilled by participants. Here, for example, it would be of value to be able to distinguish different pathways towards a more extensive gambling behavior, as the factors leading to this may be very diverse across different sub-groups of gamblers [33].

The present study had the strength of providing data from a relatively uncommon type of dataset, including detailed information about financial transactions in individuals receiving consumer credit, and therefore adds a quite novel type of data to the literature on gambling and its consequences. While the features of the current data, anonymized and impossible to link to socio-demographic or other individual characteristics, also present limitations, the size of the dataset and the objective measures of included data constitute strengths of the study.

## 6. Conclusions

In populations recruited as borrowers of money, failure to pay back loans may be more common in individuals with gambling patterns characterized as a loss of control, with short-term intense gambling episodes, and in gamblers with a higher magnitude of gambling involvement per occasion, both expressed as expenses and withdrawals. Instead, whether or not a person had any gambling at all, and the sole frequency or amount of gambling deposits, may be poor detectors of payback failure in the present kind of study sample. The present study calls for increased attention to financial problems in gamblers, and points in the direction that repeated short-term gambling deposits, and large gambling transactions per occasion, can be used as indicators of increased risk of indebtedness. Further studies should evolve from the present findings and may need to discuss interventions in consumer credit applicants with specific gambling behaviors.

## Figures and Tables

**Table 1:** Characteristics of included subjects (N = 20,750). Baseline data of primary loan occasion.

	Median	IQR (90th, 95th, 98th Percentile)	% (n)	N
***Individual characteristics***				
Number of occasions (number of loans)	1	1–3 (5, 7, 10)		20,750
Granted amount	2000	1500–3000 (5000, 7000, 10,000)		20,750
Reported income	26,000	20,000–38,000 (48,000, 55,000, 65,000)		20,750
Reported spending	10,000	6500–15,000 (19,676, 22,000, 26,000)		20,744
Reported debt	4500	0–100,000 (485,000, 1,000,000, 2,000,000)		20,749
Any gambling deposit, 30 days			29 (6024)	20,750
Any gambling deposit, 90 days			36 (7545)	20,750
Any gambling deposit, 180 days			42 (8633)	20,750
Any gambling withdrawal, 30 days			6 (1176)	20,750
Any gambling withdrawal, 90 days			9 (1923)	20,750
Any gambling withdrawal, 180 days			12 (2554)	20,750
***Extent and frequency of gambling involvement***				
Sum, gambling deposits, 30 days	0	0–205 (8293, 25,111, 52,617)		20,750
Sum, gambling deposits, 90 days	0	0–1050 (26,238, 67,084, 147,261)		20,750
Sum, gambling deposits, 180 days	0	0–2685 (49,760, 122,189, 248,146)		20,750
Sum, gambling withdrawals, 30 days	0	0–0 (0, 1000, 11,818)		20,750
Sum, gambling withdrawals, 90 days	0	0–0 (0, 9000, 38,798)		20,750
Sum, gambling withdrawals, 180 days	0	0–0 (2000, 19,498, 69,549)		20,750
Number gambling deposits, 30 days	0	0–1 (21, 50, 98)		20,750
Number gambling deposits, 90 days	0	0–5 (61, 134, 269)		20,750
Number gambling deposits, 180 days	0	0–12 (114, 242, 483)		20,750
Number gambling withdrawals, 30 days	0	0–0 (0, 1, 3)		20,750
Number gambling withdrawals, 90 days	0	0–0 (0, 3, 9)		20,750
Number gambling withdrawals, 180 days	0	0–0 (1, 5, 16)		20,750
***Intensity of gambling involvement per occasion***				
Ratio gambling deposits per 30 days	250	124–525 (1201, 1954, 3409)		6024
Ratio gambling deposits per 90 days	251	130–528 (1202, 1966, 3368)		7545
Ratio gambling deposits per 180 days	250	131–521 (1164, 1890, 3310)		8633
Ratio gambling withdrawals per 30 days	2600	1052–5646 (12,000, 20,457, 32,703)		1176
Ratio gambling withdrawals per 90 days	2662	1100–6000 (12,865, 20,286, 35,000)		1923
Ratio gambling withdrawals per 180 days	2651	1103–6000 (12,436, 20,000, 34,064)		2554

**Table 2:** Baseline correlates of an individual having a defaulted (DEF) loan on some occasion (primary loan or subsequent loans, N = 20,750). Comparisons made with Mann-Whitney U test (and one-way ANOVA) and chi-square tests for continuous and categorical variables, respectively. Numbers (n) in sub-analyses reported when other than the full sample.

	DEF (n = 5225), Median	IQR (90th, 95th, 98th Percentile)	%	Not DEF (n = 15,525), Median	IQR (90th, 95th, 98th Percentile)	%	*p* Value Continuous Variables, Mann-Whitney (ANOVA)	*p* Value Categorical Variables
***Individual characteristics***								
Number of occasions (number of loans)	1	1–2 (4, 6, 8)		1	1–3 (5, 7, 10)		<0.001 (<0.001)	
Granted amount	2500	2000–3000 (5000, 8000, 10,000)		2000	1500–3000 (5000, 7000, 10,000)		<0.001 (<0.001)	
Reported income	25,000	19,000–35,000 (45,000, 52,000, 65,000)		26,400	20,000–38,000 (48,940, 55,000, 65,506)		<0.001 *	
Reported spending	10,000	6000–13,000 (18,000, 20,000, 25,000)		10,000	7000–15000 (20,000, 22,000, 27,000, n = 15,519)		<0.001 (<0.001)	
Reported debt	3000	0–35,000 (250,000, 500,000, 1,000,000)		5000	0–125,000 (550,000, 1,200,000, 2,200,000, n = 15,524)		<0.001 *	
***Extent and frequency of gambling involvement***								
Total sum, gambling deposits, 30 days	0	0–250 (10,567, 28,324, 59,690)		0	0–200 (7728, 23,808, 50,440)		0.302 *	
Total sum, gambling deposits, 90 days	0	0–1395 (30,303, 76,109, 172,201)		0	0–1000 (25,000, 64,407, 139,922)		0.617 *	
Total sum, gambling deposits, 180 days	0	0–3697 (58,676, 135,995, 279,629)		0	0–2419 (46,739, 117,597, 240,163)		0.759 *	
Total sum, gambling withdrawals, 30 days	0	0–0 (0, 1000, 15,000)		0	0–0 (0, 1000, 11,000)		0.302 *	
Total sum, gambling withdrawals, 90 days	0	0–0 (0, 9500, 45,000)		0	0–0 (0, 8700, 37,538)		0.098 *	
Total sum, gambling withdrawals, 180 days	0	0–0 (2510, 20,322, 83,611)		0	0–0 (1947, 18,593, 65,709)		0.037 *	
Number gambling deposits, 30 days	0	0–1 (21, 53, 104)		0	0–1 (21, 49, 97)		0.520 (0.159)	
Number gambling deposits, 90 days	0	0–6 (62, 140, 289)		0	0–5 (61, 133, 260)		0.263 (0.131)	
Number gambling deposits, 180 days	0	0–13 (115, 253, 514)		0	0–11 (113, 240, 477)		0.169 (0.277)	
Number gambling withdrawals, 30 days	0	0–0 (0, 1, 3)		0	0–0 (0, 1, 3)		0.398 (0.300)	
Number gambling withdrawals, 90 days	0	0–0 (0, 3, 8)		0	0–0 (0, 3, 9)		0.508 (0.163)	
Number gambling withdrawals, 180 days	0	0–0 (1, 5, 14)		0	0–0 (1, 5, 16)		0.942 (0.197)	
***Intensity of gambling involvement per occasion***								
Ratio gambling deposits per 30 days	255	133–652 (1419, 2162, 4073, n = 1530)		242	122–500 (1135, 1868, 3012, n = 4494)		<0.001 (<0.001)	
Ratio gambling deposits per 90 days	273	138–632 (1488, 2239, 3420, n = 1932)		245	128–502 (1121, 1846, 3303, n = 5613)		<0.001 (0.004)	
Ratio gambling deposits per 180 days	268	140–620 (1410, 2129, 3636, n = 2213)		239	128–500 (1084, 1779, 3136, n = 6420)		<0.001 (0.003)	
Ratio gambling withdrawals per 30 days	3250	1432–7000 (17,128, 28,435, 56,500, n = 284)		2357	1000–5000 (10,253, 16,774, 30,000, n = 892)		<0.001 (<0.001)	
Ratio gambling withdrawals per 90 days	3487	1287–8000 (15,778, 33,405, 45,046, n = 473)		2500	1045–5500 (11,479, 18,294, 27,972, n = 1450)		<0.001 (<0.001)	
Ratio gambling withdrawals per 180 days	3487	1252–7500 (15,316, 25,148, 40,000, n = 647)		2480	1046–5500 (11,680, 18,000, 28,830, n = 1907)		<0.001 (0.001)	
***Gambling in association with loans***								
Gambling deposit prior to loan, 30 days			15			14		0.073
Gambling deposit after loan, 30 days			15			13		0.009
Gambling deposit prior to loan, 90 days			21			20		0.195
Gambling deposit after loan, 90 days			21			20		0.029
Gambling deposit prior to loan, 180 days			25			24		0.030
Gambling deposit after loan, 180 days			26			24		0.004
***Gambling patterns indicating possible loss of control***								
LOC 1000 SEK/30 days	0	0–0 (3, 8, 14)	15	0	0–0 (3, 8–14)	14	0.059 (0.418)	0.046
LOC 3000 SEK/30 days	0	0–0 (1, 5, 10)	11	0	0–0 (0, 5–10)	10	0.005 (0.060)	0.004
LOC 5000 SEK/30 days	0	0–0 (0, 3, 9)	10	0	0–0 (0, 3–7)	8	<0.001 (0.007)	<0.001
LOC 10,000 SEK/30 days	0	0–0 (0, 1, 5)	6	0	0–0 (0, 0, 3)	5	<0.001 (<0.001)	<0.001
LOC 1000 SEK/90 days	0	0–0 (8, 21, 36)	19	0	0–0 (8, 20, 35)	18	0.012 (0.429)	0.005
LOC 3000 SEK/90 days	0	0–0 (4, 13, 27)	15	0	0–0 (3, 11, 24)	13	0.002 (0.033)	0.001
LOC 5000 SEK/90 days	0	0–0 (2, 9, 21)	13	0	0–0 (1, 7, 17)	11	<0.001 (0.002)	<0.001
LOC 10,000 SEK/90 days	0	0–0 (0, 4, 11)	9	0	0–0 (0, 2, 9)	7	<0.001 (<0.001)	<0.001
LOC 1000 SEK/180 days	0	0–0 (15, 37, 60)	22	0	0–0 (14, 36, 62)	20	0.003 (0.548)	0.001
LOC 3000 SEK/180 days	0	0–0 (8, 22, 43)	18	0	0–0 (6, 21, 41)	16	<0.001 (0.082)	<0.001
LOC 5000 SEK/180 days	0	0–0 (5, 16, 33)	16	0	0–0 (3, 13, 30)	13	<0.001 (0.009)	<0.001
LOC 10,000 SEK/180 days	0	0–0 (2, 7, 18)	12	0	0–0 (0, 5, 15)	9	<0.001 (<0.001)	<0.001

LOC = loss of control. LOC calculated both as number of days in LOC state (compared as a continuous variable), and as the occurrence of any LOC (compared as a categorical variable). * ANOVA not calculated due to highly skewed data.

**Table 3:** Baseline correlates of an individual having a loan defaulted or defaulted/recovered (DEF/REC) on some occasion (primary loan or subsequent loans, N = 20,750). Comparisons made with Mann-Whitney U test (and one-way ANOVA) and chi-square tests for continuous and categorical variables, respectively. Numbers (n) in sub-analyses reported when other than the full sample.

	DEF/REC (n = 8390), Median	IQR (95th Percentile, 98th Percentile)	%	Not DEF/REC (n = 12,360), Median	IQR (90th Percentile, 95th Percentile, 98th Percentile)	%	*p* Value Continuous Variables, Mann-Whitney (ANOVA)	*p* Value Categorical Variables
***Individual characteristics***								
Number of occasions (loans)	1	1–3 (5, 6, 9)		1	1–3 (5, 7, 10)		<0.001 (<0.001)	
Granted amount	2500	2000–3000 (5000, 7000, 1000)		2000	1500–3000 (5000, 7000, 10000)		<0.001 (<0.001)	
Reported income	26,000	20,000–37,000 (47,000, 55,000, 65,000)		26,000	20,000–38,000 (48,000, 55,000, 65,000)		0.577 *	
Reported spending	10,000	6000–14,000 (18,400, 20,000, 25,000) (n = 8389)		10,000	7000–15,000 (20,000, 22,000, 27,000) (n = 12,355)		<0.001 (<0.001)	
Reported debt	3000	0–50,000 (330,000, 700,000, 1,500,000)		5000	0–130,000 (590,000, 1,250,000, 2,200,000) (n = 12,359)		<0.001 *	
***Extent and frequency of gambling involvement***								
Total sum, gambling deposits, 30 days	0	0–240 (10,360, 29,236, 59,905)		0	0–200 (7114, 21,700, 47,751)		0.040 *	
Total sum, gambling deposits, 90 days	0	0–1256 (30,171, 76,432 164,008)		0	0–979 (24,107, 60,757, 135,706)		0.225 *	
Total sum, gambling deposits, 180 days	0	0–3455 (58,318, 135,945, 273,160)		0	0–2312 (44,262, 111,758, 235,579)		0.222 *	
Total sum, gambling withdrawals, 30 days	0	0–0 (0, 445, 13,003)		0	0–0 (0, 1200, 10,912)		0.227 *	
Total sum, gambling withdrawals, 90 days	0	0–0 (0, 9540, 41,168)		0	0–0 (0, 8399, 37,390)		0.132 *	
Total sum, gambling withdrawals, 180 days	0	0–0 (1962, 19,953, 75,102)		0	0–0 (2000, 19,000, 65,985)		0.066 *	
Number gambling expenditures, 30 days	0	0–1 (21, 52, 102)		0	0–1 (21, 48, 97)		0.484 (0.217)	
Number gambling expenditures, 90 days	0	0–5 (62, 136, 278)		0	0–5 (60, 133, 260)		0.388 (0.225)	
Number gambling expenditures, 180 days	0	0–13 (116, 243, 487)		0	0–11 (112, 242, 480)		0.288 (0.453)	
Number gambling withdrawals, 30 days	0	0–0 (0, 1, 3)		0	0–0 (0, 1, 3)		0.027 (0.028)	
Number gambling withdrawals, 90 days	0	0–0 (0, 2,7)		0	0–0 (0, 3, 9)		0.138 (0.008)	
Number gambling withdrawals, 180 days	0	0–0 (1, 5, 13)		0	0–0 (1, 6, 17)		0.134 (0.009)	
***Intensity of gambling involvement per occasion***								
Ratio gambling deposits per 30 days	257	133–616 (1400, 2222, 4076, n = 2449)		233	120–494 (1069, 1651, 2837, n = 3575)		<0.001 (<0.001)	
Ratio gambling deposits per 90 days	268	137–612 (1431, 2239, 3617, n = 3074)		238	126–500 (1061, 1735, 3000, n = 4471)		<0.001 (<0.001)	
Ratio gambling deposits per 180 days	260	139–589 (1322, 2107, 3757, n = 3519)		235	125–489 (1047, 1681, 2994, n = 5114)		<0.001 (<0.001)	
Ratio gambling withdrawal per 30 days	3250	1393–7473 (19,638, 30,000, 55,900, n = 440)		2250	1000–4946 (9710, 14,148, 24,668, n = 736)		<0.001 (<0.001)	
Ratio gambling withdrawal per 90 days	3363	1340–8179 (16,341, 29,450, 46,342, n = 750)		2401	1000–4984 (10,000, 15,233, 23,914, n = 1173)		<0.001 (<0.001)	
Ratio gambling withdrawal per 180 days	3228	1258–7500 (15,478, 24,950, 40,000, n = 1001)		2413	1011–5053 (10,784, 16,126, 25,301, n = 1553)		<0.001 (<0.001)	
***Gambling in association with loans***								
Gambling deposit prior to loan, 30 days			14			14		0.464
Gambling deposit after loan, 30 days			14			13		0.052
Gambling deposit prior to loan, 90 days			21			20		0.118
Gambling deposit after loan, 90 days			21			20		0.059
Gambling deposit prior to loan, 180 days			25			23		0.003
Gambling deposit after loan, 180 days			25			23		0.005
***Gambling patterns indicating possible loss of control***								
LOC 1000 SEK/30 days	0	0–0 (3, 8, 14)	15	0	0–0 (2, 8, 14)	13	0.010 (0.150)	0.008
LOC 3000 SEK/30 days	0	0–0 (1, 5, 11)	11	0	0–0 (0, 4, 9)	10	<0.001 (0.005)	<0.001
LOC 5000 SEK/30 days	0	0–0 (0, 3, 8)	9	0	0–0 (0, 3, 7)	8	<0.001 (<0.001)	<0.001
LOC 10,000 SEK/30 days	0	0–0 (0, 1, 4)	6	0	0–0 (0, 0, 3)	4	<0.001 (<0.001)	<0.001
LOC 1000 SEK/90 days	0	0–0 (8, 20, 36)	19	0	0–0 (8, 20, 35)	17	0.003 (0.347)	0.001
LOC 3000 SEK/90 days	0	0–0 (4, 13, 26)	15	0	0–0 (3, 11, 24)	13	0.002 (0.010)	0.002
LOC 5000 SEK/90 days	0	0–0 (2, 9, 20)	13	0	0–0 (1, 7, 17)	11	<0.001 (<0.001)	<0.001
LOC 10,000 SEK/90 days	0	0–0 (0, 4, 11)	9	0	0–0 (0, 2, 9)	7	<0.001 (<0.001)	<0.001
LOC 1000 SEK/180 days	0	0–0 (16, 36, 59)	22	0	0–0 (14, 36, 62)	20	<0.001 (0.627)	<0.001
LOC 3000 SEK/180 days	0	0–0 (8, 22, 43)	18	0	0–0 (6, 20, 41)	16	<0.001 (0.048)	<0.001
LOC 5000 SEK/180 days	0	0–0 (5, 15, 32)	15	0	0–0 (3, 13, 30)	13	<0.001 (0.004)	<0.001
LOC 10,000 SEK/180 days	0	0–0 (1, 7, 17)	11	0	0–0 (0, 4, 14)	9	<0.001 (<0.001)	<0.001

LOC = loss of control. LOC calculated both as number of days in LOC state (compared as a continuous variable), and as the occurrence of any LOC (compared as a categorical variable). * ANOVA not calculated due to highly skewed data.
